# Clinicopathologic study associated with long-term survival in Japanese patients with node-negative breast cancer

**DOI:** 10.1054/bjoc.1999.0934

**Published:** 2000-01-18

**Authors:** T Kato, T Kimura, R Miyakawa, A Fujii, K Yamamoto, S Kameoka, T Nishikawa, T Kasajima

**Affiliations:** Departments of Surgery II, Surgical Pathology and Pathology, School of Medicine, Tokyo Women's Medical University, 8-1 Kawada-cho, Tokyo, 162-8666, Japan

**Keywords:** blood vessel invasion, clinical tumour size, long-term survival, node-negative breast cancer, proliferating cell nuclear antigen (PCNA)

## Abstract

This study was undertaken to determine the absolute and relative value of blood vessel invasion (BVI) using both factor VIII-related antigen and elastica van Gieson staining, proliferating cell nuclear antigen (PCNA), p53, c- *erb* B-2, and conventional prognostic factors in predicting relapse-free survival (RFS) and overall survival (OS) rates associated with long-term survival in Japanese patients with node-negative breast cancer. Two hundred patients with histological node-negative breast cancer were studied. We investigated nine clinicopathological factors, including PCNA, p53, c- *erb* B-2 using permanent-section immunohistochemistry, clinical tumour size (T), histological grade (HG), mitotic index (MI), tumour necrosis (TN), lymphatic vessel invasion (LVI) and BVI, followed for a median of 10 years (range 1–20). Twenty-one patients (10.5%) had recurrence and 15 patients (7.5%) died of breast cancer. Univariate analysis showed that BVI, PCNA, T, HG, MI, p53, c- *erb* B-2 and LVI were significantly predictive of 20-year RFS or OS. Multivariate analysis showed that BVI (*P* = 0.0159, *P* = 0.0368), proliferating cell nuclear antigen (PCNA) (*P* = 0.0165, *P* = 0.0001), and T (*P* = 0.0190, *P* = 0.0399) were significantly independent prognostic factors for RFS or OS respectively. BVI, PCNA and T were independent prognostic indicators for RFS or OS in Japanese patients with node-negative breast cancer and are useful in selecting high-risk patients who may be eligible to receive strong adjuvant therapies. © 2000 Cancer Research Campaign


*British Journal of Cancer* (2000) **82**(2), 404–411
© 2000 Cancer Research Campaign
Article no. bjoc.1999.0934

### Clinicopathologic study associated with long-term survival in Japanese patients with node-negative breast cancer

**T Kato[1], T Kimura[1], R Miyakawa[1], A Fujii[1], K Yamamoto[1], S Kameoka[1], T Nishikawa[2] and T Kasajima[3]**

Departments of [1]Surgery II, [2]Surgical Pathology and [3]Pathology, School of Medicine, Tokyo Women's Medical University, 8-1 Kawada-cho, Shinjuku-ku, Tokyo, 162-8666, Japan

**Summary** This study was undertaken to determine the absolute and relative value of blood vessel invasion (BVI) using both factor VIII-related antigen and elastica van Gieson staining, proliferating cell nuclear antigen (PCNA), p53, c-*erb*B-2, and conventional prognostic factors in predicting relapse-free survival (RFS) and overall survival (OS) rates associated with long-term survival in Japanese patients with node-negative breast cancer. Two hundred patients with histological node-negative breast cancer were studied. We investigated nine clinicopathological factors, including PCNA, p53, c-*erb*B-2 using permanent-section immunohistochemistry, clinical tumour size (T), histological grade (HG), mitotic index (MI), tumour necrosis (TN), lymphatic vessel invasion (LVI) and BVI, followed for a median of 10 years (range 1–20). Twenty-one patients (10.5%) had recurrence and 15 patients (7.5%) died of breast cancer. Univariate analysis showed that BVI, PCNA, T, HG, MI, p53, c-*erb*B-2 and LVI were significantly predictive of 20-year RFS or OS. Multivariate analysis showed that BVI ($P$ = 0.0159, $P$ = 0.0368), proliferating cell nuclear antigen (PCNA) ($P$ = 0.0165, $P$ = 0.0001), and T ($P$ = 0.0190, $P$ = 0.0399) were significantly independent prognostic factors for RFS or OS respectively. BVI, PCNA and T were independent prognostic indicators for RFS or OS in Japanese patients with node-negative breast cancer and are useful in selecting high-risk patients who may be eligible to receive strong adjuvant therapies. © 2000 Cancer Research Campaign

**Keywords:** blood vessel invasion; clinical tumour size; long-term survival; node-negative breast cancer; proliferating cell nuclear antigen (PCNA)

There is now evidence that adjuvant therapy is useful in breast carcinomas without axillary lymph-node metastases (Mansour et al, 1989; Fisher et al, 1996). Various histological features have been proposed as prognostic factors (Davis et al, 1986; van de Velde et al, 1986; Lee et al, 1990; Clayton et al, 1991; Clemente et al, 1992; Gasparini et al, 1994; Kato et al, 1996; O'Malley et al, 1996), but no indicators for the need of adjuvant treatment have yet been clearly established. Some studies have suggested that peritumour lymphatic and blood vessel invasion (PLBI) (Lee et al, 1990; de Mascarel et al, 1998; Lampe et al, 1998) or peritumour lymphatic vessel invasion (LVI) (Clemente et al, 1992; Gasparini et al, 1994; Lauria et al, 1996) are significant prognostic factors. However, there are few studies of the prognostic significance of peritumour blood vessel invasion (BVI) (Gasparini et al, 1994; Kato et al, 1996). It was more difficult to identify invasion of small vessels and capillaries and the larger vessels surrounded by elastic tissue in breast cancer in routine haematoxylin and eosin (H&E)-stained tissue sections than in gastric or colorectal cancer. A wide range of frequencies has been reported for BVI among patients with breast cancer. The differences may be due to various methods, interobserver discordance, and the characteristics of different series, but the prognostic significance of BVI remains controversial (Sears et al, 1982; Weigand et al, 1982; van de Velde et al, 1986; Lauria et al, 1996).

This study was undertaken to determine the absolute and relative value of BVI using both factor VIII-related antigen and elastica van Gieson staining, p53, PCNA, c-*erb*B-2 which are relatively new prognostic factors, and conventional prognostic factors in predicting relapse-free survival (RFS) and overall survival (OS) rates with long-term follow-up in Japanese patients with node-negative breast cancer.

### PATIENTS AND METHODS

### Patients

Data for this study were collected from 200 selected node-negative breast cancer patients operated on between 1971 and 1987 at the Tokyo Women's Medical University Hospital for whom we had sufficient clinical and pathologic material to determine all the biological markers. The distribution of clinical and pathologic data for the entire patient population is listed in Table 1. It shows the main clinicopathologic characteristics of the 200 node-negative assessable patients. Eighty-three of these patients (41.5%) were treated with radical mastectomy, 76 patients (38.0%) with modified radical mastectomy, and 41 patients (20.5%) with extended radical mastectomy. One hundred and fifty-two of them (76.0%) were treated by chemotherapy. Forty-one (20.5%) were treated by mitomycin C (mitomycin C was given intravenously after mastectomy, total doses ranged from 32 mg to 40 mg) and 111 patients

*Received 7 August 1998*
*Revised 14 July 1999*
*Accepted 15 July 1999*

*Correspondence to:* T Kato

**Table 1** Clinicopathologic characteristics of assessable node-negative patients

| Characteristics | No. of patients | % |
|---|---|---|
| Total eligible | 200 | |
| Age, years | | |
| Median | 48 | |
| Range | 15–82 | |
| Survival follow-up, years | | |
| Median | 10 | |
| Range | 1–20 | |
| Local recurrence | 21 | 10.5 |
| Deaths | 15 | 7.5 |
| Tumour size, T | | |
| T1 | 129 | 64.5 |
| T2 | 56 | 28.0 |
| T3 | 15 | 7.5 |
| Menopausal status | | |
| Pre | 96 | 48.0 |
| Post | 94 | 47.0 |
| Unknown | 10 | 5.0 |
| Blood vessel invasion | | |
| – | 166 | 83.0 |
| + | 34 | 17.0 |
| Lymphatic vessel invasion | | |
| – | 174 | 87.0 |
| + | 26 | 13.0 |
| Histological grade (HG) | | |
| HG I (Well) | 98 | 49.0 |
| HG II (Moderate) | 55 | 27.5 |
| HG III (Poor) | 47 | 23.5 |
| Mitotic grade | | |
| (Mitotic figures per 10 HPF, ×400) | | |
| 0 (Grade I) | 97 | 48.5 |
| 1–4 (Grade II) | 41 | 20.5 |
| ≥5 (Grade III) | 62 | 31.0 |
| Tumour necrosis | | |
| – | 111 | 55.5 |
| + | 69 | 34.5 |
| Histological classification | | |
| Infiltrating ductal carcinoma | 180 | 90.0 |
| Infiltrating lobular carcinoma | 11 | 5.5 |
| Medullary carcinoma | 3 | 1.5 |
| Mucinous carcinoma | 6 | 3.0 |
| PCNA | | |
| – | 155 | 77.5 |
| + | 38 | 19.0 |
| NA | 7 | 3.5 |
| p53 | | |
| – | 155 | 77.5 |
| + | 43 | 21.5 |
| NA | 5 | 2.5 |
| c-*erb*B-2 | | |
| – | 149 | 74.5 |
| + | 42 | 21.0 |
| NA | 9 | 4.5 |

PCNA, proliferating cell nuclear antigen; NA, not assessable.

(55.5%) received 5-fluorouracil or its derivatives orally. The median follow-up duration of the patients was 10 years (range 1–20).

### Pathological studies

The original histologic sections of biopsy and mastectomy specimens were reviewed. Paraffin-embedded tissue samples of 4.5-μm-thick sections stained with H&E were histopathologically assessed. The pathologic material was reviewed without any knowledge of the eventual clinical outcome. Conventional clinicopathologic features were observed and recorded, including the clinical tumour size, histological classification, histological grade, mitotic grade, LVI and tumour necrosis. The clinical tumour size was determined based on the TNM classification, the histological grade was decided based on the Bloom–Richardson grade (Bloom and Richardson, 1957), and LVI was determined based on H&E staining. The mitotic grade was divided into three grades based on the number of mitoses in 10 high power fields (×400): Grade I showed no mitoses, Grade II 1–4 mitoses, and Grade III 5 or more mitoses. Sections of the breast tumour that were stained with H&E were used to select the maximal area of all the cut surfaces of the tumour that included the invasive component.

### Immunocytochemical determinations

### *PCNA*

The 4.5-μm thickness sections were dewaxed in xylene and rehydrated in decreasing concentrations of ethanol. The sections were then brought to distilled water. The endogenous peroxidase activity was blocked with 0.3% of hydrogen peroxide for 20 min. The sections were washed in 0.05 M Tris-buffered saline, and treated with a protein blocking agent (Japan Tanner/Lipshaw Corporation, USA) before staining to reduce non-specific antibody binding. Immunostaining was performed using a mouse monoclonal anti-human-PCNA antibody (PC10, Novocastra Laboratories, UK) diluted at 1:100 for 24 h at 4°C. Antibody binding was visualized by the streptavidin–biotin–immunoperoxidase method (Omnitags kit; Japan Tanner/Lipshaw Corporation, USA), followed by 0.05% 3,3′-diaminobenzidine tetrahydrochloride development and counterstained with Meyer’s haematoxylin and mounted. A normal human tonsil served as a positive control. As an internal negative control, each tumour was incubated with non-immune mouse immunoglobulin G (lgG) instead of the primary antibody, followed by the procedure described above.

The growth fraction by PCNA staining was evaluated by counting 500 consecutive cells on one slide per tumour with a ×400 magnification and an index of positive cells to total number of cells (labelling index [LI]) was made. LI was evaluated and scored as (–) (< mean of LI) and (+) (⩾ mean of LI).

### *p53*

After routine deparaffinization, the sections were pretreated by microwaving at 500 W in 10 mM citrate buffer (pH 6) as an antigen retrieval. The slides were placed in citrate buffer and microwaved at full power for 5 min. After cooling by running them through cold water, they were then slowly brought to distilled water. The immunostaining of p53 was performed using a rabbit polyclonal anti-p53 antibody (CM1, Novocastra Laboratories, UK) diluted at 1:100 for 24 h at 4°C. The remaining steps for immunostaining were the same as described earlier. Negative controls consisted of omission of the primary antibody. Positive controls were sections of colon carcinomas known to have p53 gene mutations and p53 protein accumulation.

As above, the growth fraction by p53 protein staining was evaluated by counting 500 consecutive cells and LI was made. LI was evaluated and scored as (–) (< mean of LI) and (+) (⩾ mean of LI).

© 2000 Cancer Research Campaign 

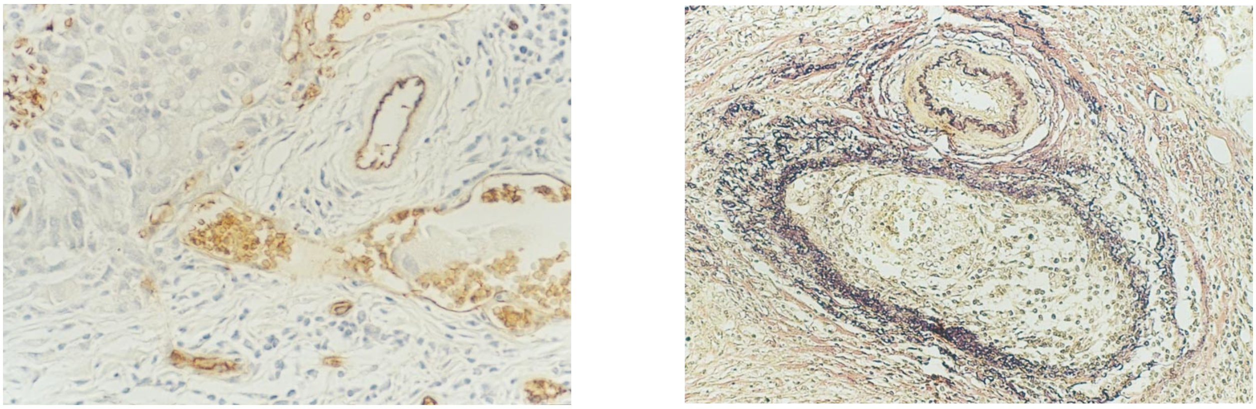

**Figure 1** (**A**) Infiltrating duct carcinoma of breast, showing tumour cells within the lumen of a small blood vessel for Factor VIII-related antigen (haematoxylin counter stain, ×400). (**B**) Clusters of neoplastic cells were identified within the lumen of a blood vessel and were seen growing near the external elastic membrane stained by elastica van Gieson (elastica van Gieson staining, ×200)

### *c*-erb*B-2*

Staining for the c-*erb*B-2 protein was performed using a rabbit polyclonal anti-human c-*erb*B-2 protein antibody (Dako, Copenhagen, Denmark) diluted at 1:100 for 24 h at 4°C, using the same method as for the p53 protein. Positive controls included breast carcinomas known to exhibit high levels of protein. Negative controls were obtained by omission of the primary antibody. For c-*erb*B-2 protein expression, membrane staining in at least 50% of the tumour cells were considered positive according to the criteria of Wright et al (1989).

### *BVI*

One representative section per tumour was stained by using a monoclonal antibody (von Willebrand factor F8/86, Dako, Copenhagen, Denmark) with the use of a standard immunoperoxidase technique and by elastica van Gieson staining described previously (Kato et al, 1996). Briefly, formalin-fixed, paraffin-embedded 4.5-μm sections were dewaxed in xylene, rehydrated and immunostaining was performed using a monoclonal antibody diluted at 1:200 at 37°C for 1 h.

BVI was determined using factor VIII-related antigen staining and elastica van Gieson staining (Figure 1). The distinction between lymphatic vessels and blood vessels is sometimes difficult to determine and arbitrary. While endothelial cells in the lymphatic vessels were occasionally stained, the pattern of staining in the lymphatic vessels was very faint, discontinuous and inconsistent, in contrast to the intense and continuous staining observed in the vascular endothelium. On this basis, blood vessels could be differentiated from lymphatic vessels in our study. All doubtful cases were considered to be negative.

BVI detected by elastica van Gieson staining was defined by criteria similar to those of Weigand et al (1982). Blood vessels were identified by the following characteristics. Erythrocytes in the lumen, an endothelial cell lining, and the presence of elastic tissue around large vessels, and ducts were identified by a zone of acidophilic non-elastic tissue inside the elastic ring surrounding the ducts. When BVI was difficult to distinguish from ducts invaded by carcinoma in situ, the case was considered to be negative.

### Statistical analysis

The statistical analysis of the data was performed with the Survival Tools for Statview-J 4.5 package (Abacus Concepts, Berkeley, CA, USA) on an Apple Power Macintosh 8100/100AV. We examined the univariate relationships between prognostic indicators and RFS and OS by fitting Kaplan–Meier survival curves (Kaplan and Meier, 1958) to various levels of the prognostic indicators. We then looked for differences among the curves using the logrank test (Mantel, 1966). For the multivariate analysis, the Cox proportional hazards regression model was also used (Cox, 1972).

### RESULTS

### Clinical outcome

Among the 200 node-negative patients, there were 21 tumour-related local recurrences and 15 tumour-related deaths. The probability of 5-year RFS and OS in the total patient population was 91.9% and 95.0%, respectively. However, that of 20-year RFS and OS was 88.7% and 91.3% respectively. In addition, there were seven patients who were lost in the follow-up, and 165 patients who are alive and 13 patients who died of unrelated causes without recurrent tumours, with follow-up examinations of up to 20 years (median 10 years).

### Univariate analysis

The prognostic factors found to be significantly associated with 20-year RFS were PCNA ($P < 0.0001$), clinical tumour size ($P < 0.0001$), BVI ($P < 0.0001$), histological grade ($P = 0.0007$), mitotic grade ($P = 0.0016$), p53 ($P = 0.0043$), c-*erb*B-2 ($P = 0.0451$) and LVI ($P = 0.0467$) (Table 2 and Figures 2A, 3A and 4A). Tumour necrosis was not associated with RFS. Moreover, PCNA ($P < 0.0001$), clinical tumour size ($P < 0.0001$), BVI ($P < 0.0001$), histological grade ($P = 0.0016$), mitotic grade ($P = 0.0053$), LVI ($P = 0.009$), p53 ($P = 0.0095$), c-*erb*B-2 ($P = 0.0144$) and tumour necrosis ($P = 0.045$) were shown to be significantly associated with 20-year OS (Table 3 and Figures 2B, 3B and 4B).

### Multivariate analysis

Multivariate analysis showed that BVI was the strongest independent prognostic factor for RFS ($\chi^2 = 5.810$; $P = 0.0159$; Table 4). Patients with positive BVI were 3.48 times more likely to relapse than those with negative BVI. PCNA was the second most predictive independent prognostic factor ($\chi^2 = 5.745$; $P = 0.0165$). The

© 2000 Cancer Research Campaign

**Table 2** Univariate analysis of the value of prognostic factors for RFS

| Variable | RR[a] | 95% Confidence interval | $\chi^2$ | *P* |
|---|---|---|---|---|
| Blood vessel invasion | | | | |
| – vs + | 6.365 | 2.697–15.02 | 17.85 | <0.0001 |
| PCNA | | | | |
| – vs + | 9.193 | 3.7–22.8 | 22.92 | <0.0001 |
| Tumour size, T | | | | |
| T1 vs T2 | 2.868 | 1.038–7.920 | 4.133 | 0.0421 |
| T1 vs T3 | 8.99 | 3.016–26.80 | 15.53 | <0.0001 |
| Histological grade (HG) | | | | |
| HG I vs HG II | 2.456 | 0.659–9.149 | 1.793 | 0.1806 |
| HG I vs HG III | 7.167 | 2.309–22.25 | 11.61 | 0.0007 |
| Mitotic grade | | | | |
| Grade I vs Grade II | 7.697 | 1.553–38.15 | 6.246 | 0.0125 |
| Grade I vs Grade III | 11.03 | 2.489–48.91 | 9.99 | 0.0016 |
| p53 | | | | |
| – vs + | 3.489 | 1.481–8.219 | 8.173 | 0.0043 |
| Lymphatic vessel invasion | | | | |
| – vs + | 3.023 | 1.016–8.993 | 3.955 | 0.0467 |
| c-*erb*B-2 | | | | |
| – vs + | 2.496 | 1.020–6.108 | 4.016 | 0.0451 |
| Tumour necrosis | | | | |
| – vs + | 2.307 | 0.978–5.441 | 3.649 | 0.0561 |

[a] Hazards ratio from Cox regression analysis. RFS, relapse-free survival; PCNA, proliferating cell nuclear antigen; RR, relative risk.

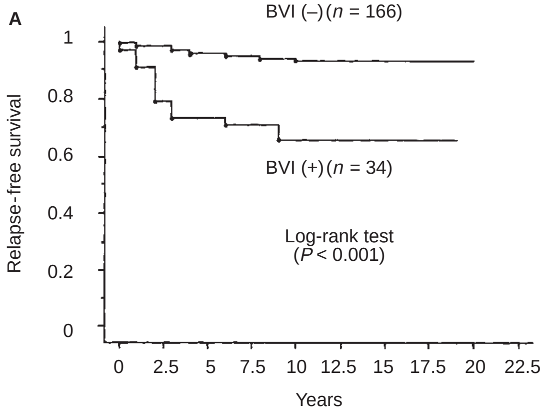

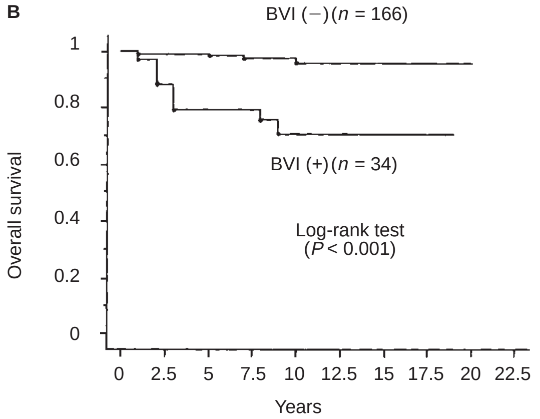

**Figure 2** Kaplan–Meier survival curve for node-negative patients. (**A**) Relapse-free survival stratified by BVI. (**B**) Overall survival related to BVI

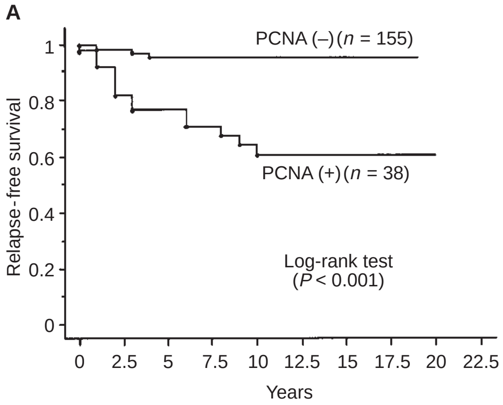

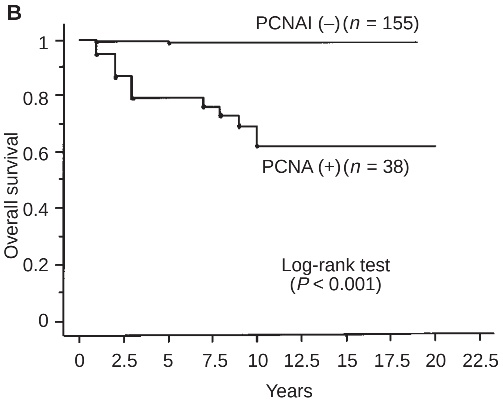

**Figure 3** Kaplan–Meier survival curve for node-negative patients. (**A**) Relapse-free survival stratified by PCNA. (**B**) Overall survival related to PCNA

third most significant and independent prognostic factor was clinical tumour size ($\chi^2$ = 5.498; *P* = 0.0190).

For OS, PCNA was the strongest independent predictor ($\chi^2$ = 15.46; *P* < 0.0001). The relative risk (RR) of death was 14.15 times higher in those with positive PCNA than in those with negative PCNA. The second and third significant and independent prognostic factors were BVI and clinical tumour size ($\chi^2$ = 4.361, *P* = 0.0368; $\chi^2$ = 4.222, *P* = 0.0399 respectively). Histological grade, mitotic grade, p53, LVI, tumour necrosis and c-*erb*B-2 failed to retain an independent and significant value of RFS or OS in multivariate analysis.

### Characteristics of the treatment

We examined the effect of treatment in node-negative patients for RFS and OS. The modality of operation was not associated with either RFS or OS. However, chemotherapy was significantly associated with OS but not with RFS ($\chi^2$ = 4.471, *P* = 0.0345; $\chi^2$ = 1.230, *P* = 0.2674 respectively). We fitted a model including three prognostic factors of PCNA, BVI and chemotherapy, and compared them by multivariate analysis. PCNA and BVI were the significant and independent factors ($\chi^2$ = 14.291, *P* = 0.0002; $\chi^2$ = 6.565, *P* = 0.0104 respectively), but chemotherapy was not an independent predictor for OS ($\chi^2$ = 0.128, *P* = 0.7204).

© 2000 Cancer Research Campaign

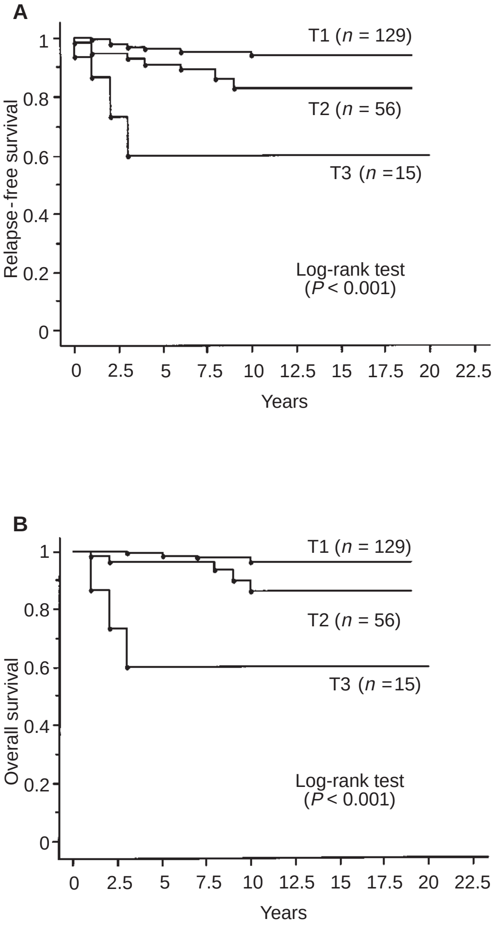

**Figure 4** Kaplan–Meier survival curve for node-negative patients. (**A**) Relapse-free survival stratified by clinical tumour size. (**B**) Overall survival related to clinical tumour size

**Table 3** Univariate analysis of the value of prognostic factors for OS

| Variable | RR[a] | 95% Confidence interval | $\chi^2$ | *P* |
|---|---|---|---|---|
| Blood vessel invasion | | | | |
| – vs + | 7.277 | 2.703–19.60 | 15.43 | <0.0001 |
| PCNA | | | | |
| – vs + | 19.37 | 5.516–68.03 | 21.39 | <0.0001 |
| Tumour size, T | | | | |
| T1 vs T2 | 2.557 | 0.739–8.849 | 2.198 | 0.1382 |
| T1 vs T3 | 12.59 | 3.832–41.33 | 17.43 | <0.0001 |
| Histological grade (HG) | | | | |
| HG I vs HG II | 1.967 | 0.397–9.752 | 0.686 | 0.4076 |
| HG I vs HG III | 7.979 | 2.192–29.04 | 9.929 | 0.0016 |
| Mitotic grade | | | | |
| Grade I vs Grade II | 9.983 | 1.115–89.35 | 4.234 | 0.0396 |
| Grade I vs Grade III | 18.4 | 2.375–142.6 | 7.775 | 0.0053 |
| p53 | | | | |
| – vs + | 3.661 | 1.373–9.761 | 6.731 | 0.0095 |
| Lymphatic vessel invasion | | | | |
| – vs + | 4.534 | 1.459–14.09 | 6.83 | 0.009 |
| c-*erb*B-2 | | | | |
| – vs + | 3.698 | 1.297–10.544 | 5.984 | 0.0144 |
| Tumour necrosis | | | | |
| – vs + | 2.754 | 1.023–7.417 | 4.017 | 0.045 |

[a] Hazards ratio from Cox regression analysis. OS, overall survival; PCNA, proliferating cell nuclear antigen; RR, relative risk.

### DISCUSSION

Results of the present study indicate that BVI, PCNA and clinical tumour size are independent prognostic indicators of RFS and OS, and are useful in selecting high-risk node-negative breast cancer patients who may be eligible to receive strong adjuvant therapies. However, p53, c-*erb*B-2 and other conventional prognostic factors were not associated with the outcome of the multivariate analysis.

There is adequate evidence that adjuvant chemotherapy improves survival among patients without axillary-lymph-node metastases (Mansour et al, 1989; Fisher et al, 1996). There have been many reports about the poor prognostic factors indicating the need for adjuvant treatment in node-negative patients other than BVI, PCNA, tumour size. LVI (van de Velde et al, 1986; Lee et al, 1990; Clemente et al, 1992; Gasparini et al, 1994; Lauria et al, 1996), angiogenesis (Gasparini et al, 1994, Eppenberger et al, 1998), the mitotic count (Clayton et al, 1991), the Bloom–Richardson grade (Davis et al, 1986, Iwaya et al, 1997), p53 (Gasparini et al, 1994, Dittadi et al, 1997; Molina et al, 1998), c-*erb*B-2 (O'Malley et al, 1996; Andrulis et al, 1998) and vascular endothelial growth factor (Gasparini et al, 1997) have all been reported as significant prognostic factors. However, no indicators for the need of adjuvant treatment have yet been clearly established. This study was undertaken to determine the absolute and relative value of BVI using both factor VIII-related antigen and elastica van Gieson staining, p53, PCNA, c-*erb*B-2 which are relatively new prognostic factors, and conventional prognostic factors

**Table 4** Multivariate analysis showing independent factors of RFS and OS

| Variable | RR | RFS[a] 95% Confidence interval | $\chi^2$ | *P* | RR | OS[b] 95% Confidence interval | $\chi^2$ | *P* |
|---|---|---|---|---|---|---|---|---|
| Blood vessel invasion | | | | | | | | |
| – vs + | 3.48 | 1.262–9.592 | 5.81 | 0.0159 | 3.211 | 1.074–9.598 | 4.361 | 0.0368 |
| PCNA | | | | | | | | |
| – vs + | 3.807 | 1.276–11.36 | 5.745 | 0.0165 | 14.15 | 3.776–53.00 | 15.46 | <0.0001 |
| Tumour size, T | | | | | | | | |
| T1 vs T2 | 2.036 | 0.692–5.986 | 1.669 | 0.1964 | 0.873 | 0.251–3.034 | 0.046 | 0.831 |
| T1 vs T3 | 4.068 | 1.259–13.15 | 5.498 | 0.019 | 3.86 | 1.064–14.00 | 4.222 | 0.0399 |

Shown is the multivariate analysis employing Cox proportional-hazard regression of prognostic factors of RFS and OS. [a] In the model regarding RFS three prognostic indicators are compared. [b] In the model regarding OS three prognostic indicators are compared. RFS, relapse-free survival; OS, overall survival; PCNA, proliferating cell nuclear antigen; RR, relative risk.

*© 2000 Cancer Research Campaign*

in predicting RFS and OS rates with long-term follow-up in Japanese patients with node-negative breast cancer.

Weigand et al (1982) and others (Sampat et al, 1978) reported that the presence of tumour cell (TC) emboli in blood vessels correlated significantly with a high rate of recurrence. However, it was more difficult to identify invasion of small vessels and capillaries in breast cancer in routine H&E-stained tissue sections than in gastric cancer or in colorectal cancer. Therefore we used factor VIII-related antigen staining and were thus able to successfully detect BVI. Factor VIII-related antigen has been well established as a marker for vascular endothelial cells (Bettelheim et al, 1984; Martin et al, 1987), and is consistently found in normal endothelial cells in blood vessels; while endothelial cells in lymphatic vessels were occasionally stained, the pattern of staining was very faint and discontinuous. Several studies (Bettelheim et al, 1984; Martin et al, 1987) have examined the use of immunohistochemical techniques to identify blood vessels and lymphatic vessels; but others (Saigo et al, 1987) have reported that immunohistochemical stains may be of assistance in occasional cases and that examination of H&E stains is the most reliable method.

Some clinical studies suggested that to use an antibody to CD31 may be superior to using factor VIII-related antigen (Horak et al, 1992; Fox et al, 1994); however, the other study reported that this greater sensitivity of anti-CD31 of vascular endothelium did not yield results more discriminating for predicting survival outcome than results produced with factor VIII-related antigen (Gasparini et al, 1994). It may lack accuracy, however, many stromal vessels can be stained very well and be counted by factor VIII-related antigen staining as we previously reported and there was a strong relationship between BVI and angiogenesis (Kato et al, 1999). In this study, incidence of BVI detected by factor VIII-related antigen staining was about twice as high as that of BVI detected by elastica van Gieson staining, so factor VIII-related antigen is a practical and effective way of identifying endothelial cells lining arteries, veins and capillaries.

In our experience, it was difficult to distinguish among BVI, pseudoemboli resulting from shrinkage where cancer cells peeled off from the ducts by poor fixing, and lymphatic invasion only by H&E staining, while it was relatively easy to detect BVI using factor VIII-related antigen staining. Immunohistochemical staining with factor VIII-related antigen staining facilitated the identification of blood vessels and lymphatic vessels and also helped to eliminate pseudoemboli resulting from shrinkage. Detection of the blood vessels infiltrated by tumour cells and those in which tumour cells were floating was easy, however, detection of the blood vessels filled with TC emboli by this staining alone was difficult as we have previously published (Kato et al, 1996). There was possibility of overlooking the blood vessels filled with TC emboli using factor VIII-related antigen staining alone.

Therefore we also used elastica van Gieson staining to detect the blood vessels with TC emboli. To actually detect blood vessels filled with TC emboli and invaded by cancer cells by factor VIII-related antigen staining alone was impossible because the endothelial cells could not be stained well. Elastica van Gieson staining proved to be a useful method for such blood vessels filled with TC emboli. However, it was sometimes difficult to distinguish BVI from ductal carcinoma in situ covered with elastic fibre. In such cases, it was considered negative. We used factor VIII-related antigen staining to detect the small blood vessels which did not have elastic fibre and also used elastica van Gieson staining to detect the large blood vessels filled with TC emboli, and thereby blood vessels detected by the former staining method were differentiated from those detected by the latter. We therefore consider it necessary to use both staining methods when we detect BVI.

In this study BVI in the 200 node-negative patients who we observed was seen in 34 cases (17.0%). This rate of BVI is higher than that of another study by Lee et al (1990). When they examined the prognostic value of PLBI, the frequencies of BVI and LVI were 7% and 21% respectively. They detected BVI using factor VIII-related antigen staining and ABH isoantigen. As we could detect the larger blood vessels filled with TC emboli by elastica van Gieson staining, our frequencies of BVI might be higher than theirs. However, when PLBI was separated into LVI and BVI individually, the prognostic importance of TC emboli was retained in both groups. Our results are similar to theirs regarding survival. BVI is thus suggested to be an adverse prognostic factor among node-negative patients.

A relationship between tumour size and prognosis has been reported in several studies of node-negative patients. In some large series, it was found that increasing tumour size was related to the likelihood of recurrence (Rosen et al, 1989; Clayton et al, 1991; Lampe et al, 1998). But in several other studies, tumour size was not found to be significant (Sears et al, 1982; van de Velde et al, 1986). In the present study, clinical tumour size was an effective factor. The T3 tumour had a particularly poor prognosis.

Cell proliferation is an important prognostic indicator in breast cancer (McGuire et al, 1990). The proliferative index of breast cancer has been judged by immunohistochemistry with monoclonal antibodies to other proliferation markers such as PCNA and Ki-67 (Isola et al, 1990; Bianchi et al, 1993; Sitonen et al, 1993; Railo et al, 1997; Clahsen et al, 1998). They have been shown to have some prognostic value in breast cancer. In this study, PCNA was shown to be significantly associated with RFS and OS. PCNA was the strongest independent factor for OS. The relative risk of death was 14.2 times higher in patients with positive PCNA than in those with negative PCNA proving it a strong prognostic factor among node-negative patients.

There are contradictory findings concerning the correlation of c-*erb*B-2 expression and survival of patients in node-negative breast cancer patients; some clinical trials confirmed this association (O'Malley et al, 1996; Andrulis et al, 1998), whereas others found no correlation (Harbeck et al, 1998; Lampe et al, 1998). In this study, c-*erb*B-2 expression was a significant prognostic factor in univariate analysis, however, not associated with the outcome of the multivariate analysis. These discrepancies may be due to some technical reasons, the small number of cases studied, the different follow-up period, the different races and different characteristics of the patients.

The association of p53 protein accumulation and prognosis in node-negative breast cancer patients has been alternately demonstrated. Some clinical studies found correlation between p53 protein accumulation and prognosis (Gasparini et al, 1994; Dittadi et al, 1997; Molina et al, 1998); however, other authors did not find any correlation at all (Harbeck et al, 1998; Lampe et al, 1998). The prognostic value remains controversial. In our sample, p53 has a prognostic role in univariate analysis and was a borderline significant prognostic factor in multivariate analysis for 20-year RFS (RR = 2.506, 95% confidence interval = 0.95–6.606, $P = 0.0633$). The significance of p53 as a prognostic role in breast cancer should be addressed with methods that investigate the correlation

© 2000 Cancer Research Campaign of other new biological factors which warrants further and larger studies.

Seventy-six per cent of all node-negative breast cancer patients were treated by mitomycin C or 5-fluorouracil or its derivatives in this study. The percentage of patients who received chemotherapy is quite higher than that in recent studies of other researchers (Barbareschi et al, 1996). We compared the prognosis of the node-negative patients who underwent chemotherapy with those who did not. Our study confirms that this kind of weak regimen of chemotherapy appears somewhat effective in node-negative patients for OS by univeriate analysis but not for RFS. When we compared PCNA, BVI and chemotherapy by multivariate analysis, such a regimen was not an independent factor for OS, so it was not relevant as a significant prognostic factor. In the same way, there was no effectiveness of modality of operation for OS nor RFS.

The results of our study shows that the probability of 5-year RFS and OS in the total patient population was better than that of other researchers (Tandon et al, 1990; Gasparini et al, 1994). This may be due to various factors. First, there is different epidemiology, especially a higher proportion of Japanese patients in the premenopausal age group than the Caucasian women in Haagensen's book, and the distribution by age at the time of the operation in Japanese patients was different from that in those Caucasian patients (Haagensen, 1986). Second, the prognosis of Japanese patients is better than that of Caucasian women due to biological behaviour (Wynder et al, 1963; Stemmermann et al, 1985). Finally, there were more patients in this study treated by chemotherapy than those in other studies (Barbareschi et al, 1996).

We acknowledge that the number of patients in some of the subgroups in our analysis is not as large as would be desirable and that a larger number of patients will be required to better assess these relationships with greater certainty, but we concluded that BVI, PCNA and clinical tumour size are prognostic factors of RFS and OS in Japanese patients with node-negative breast cancer.

### ACKNOWLEDGEMENTS

The authors thank Mr Robert E Brandt for his help editing the manuscript.

### REFERENCES

Andrulis IL, Bull SB, Blackstein ME, Sutherland D, Mak C, Sidlofsky S, Pritzker KP, Hartwick RW, Hanna W, Lickley L, Wilkinson R, Qizilbash A, Ambus U, Lipa M, Weizel H, Katz A, Baida M, Mariz S, Stoik G, Dacamara P, Strongitharm D, Geddie W and McCready D (1998) neu/erbB-2 amplification identifies a poor-prognosis group of women with node-negative breast cancer. Toronto Breast Cancer Study Group. *J Clin Oncol* **16**: 1340–1349

Barbareschi M, Caffo O, Veronese S, Leek RD, Fina P, Fox S, Bonnzanini M, Girlando S, Morelli L, Eccher C, Pezzella F, Doglioni C, Palma PD and Harris A (1996) Bcl-2 and p53 expression in node-negative breast carcinoma: a study with long-term follow-up. *Hum Pathol* **27**: 1149–1155

Bettelheim R, Mitchell D and Gusterson BA (1984) Immunocytochemistry in the identification of vascular invasion in breast cancer. *J Clin Pathol* **37**: 364–366

Bianchi S, Paglieranti M, Zampi G, Cardona G, Cataliotti L, Bonardi R, Zappa M and Ciatto S (1993) Prognostic value of proliferating cell nuclear antigen in lymph node-negative breast cancer patients. *Cancer* **72**: 120–125

Bloom HIJ and Richardson WW (1957) Histologic grading and prognosis. *Br J Cancer* **11**: 359–377

Clahsen PC, van de Velde CJ, Duval C, Pallud C, Mandard AM, Delobelle-Deroide A, van den Broek L, Sahmound TM and van de Vijver MJ (1998) P53 protein accumulation and response to adjuvant chemotherapy in premenopausal women with node-negative early breast cancer. *J Clin Oncol* **16**: 470–479

Clayton F (1991) Pathologic correlates of survival in 378 lymph node-negative infiltrating ductal breast carcinomas. Mitotic count is the best single predictor. *Cancer* **68**: 1309–1317

Clemente CG, Boracchi P, Andreola S, Del Vecchio M, Veronesi P and Rilke FO (1992) Peritumoral lymphatic invasion in patients with node-negative mammary duct carcinoma. *Cancer* **69**: 1396–1403

Cox DR (1972) Regression models and life tables. *J. R. Stat.Soc* **B 34**: 187–202

Davis BW, Gelber RD, Goldhirsch A, Hartmann WH, Locher GW, Reed R, Golouh R, Save-Soderbergh J, Holloway L, Russel I and Rudenstam M (1986) Prognostic significance of tumor grade in clinical trials of adjuvant therapy for breast cancer with axillary lymph-node metastasis. *Cancer* **58**: 2662–2670

de Mascarel I, Bonichon F, Durand M, Mauriac L, MacGrogan G, Soubeyran I, Picot V, Avril A, Coindre JM and Trojani M (1998) Obvious peritumoral emboli: an elusive prognostic factor reappraised. Multivariate analysis of 1320 node-negative breast cancers. *Eur J Cancer* **34**: 58–65

Dittadi R, Brazzale A, Mione R, Di Fresco S, Gatti C, Vinante O, Bassan F, Nascimben O and Gion M (1997) Quantitative chemiluminescent immunoassay of p53: prognostic significance in 220 node-negative breast cancer tissue. *Anticancer Res* **17**: 4691–4696

Eppenberger Kueng W, Schlaeppi JM, Roesel JL, Benz C, Mueller H, Matter A, Zuber M, Luescher K, Litschgi M, Schmitt M, Foekens JA and Eppenberger-Castori S (1998) Marker of tumor angiogenesis and proteolysis independently define high- and low-risk subsets of node-negative breast cancer patients. *J Clin Oncol* **16**: 3129–3136

Fisher B, Dignam J, Mamounas EP, Castantino JP, Wickerham DL, Redmond C, Wolmark N, Dimitrov NV, Bowman DM, Glass AG, Atkins JN, Abramson N, Sutherland CM, Aron BS and Margolese RG (1996) Sequential methotrexate and fluorouracil for the treatment of node-negative breast cancer patients with estrogen-receptor-negative tumors: eight-year results from national surgical adjuvant breast and bowel project (NSAB) B-13 and first report of findings from NSABP B-19 comparing methotrexate and fluorouracil with conventional cyclophosphamide, methotraxate, and fluorouracil. *J. Clin Oncol* **14**: 1982–1992

Fox SB, Leek RD, Smith K, Hollyer J, Greenall M and Harris AL (1994) Tumor angiogenesis in node-negative breast carcinomas – relationship with epidermal growth factor receptor, estrogen receptor, and survival. *Breast Cancer Res Treat* **29**: 109–116

Gasparini G, Weidner N, Bevilacqua P, Maluta S, Palma PD, Caffo O, Barbareshi M, Baracchi P, Marubini E and Pozza F (1994) Tumor microvessel density, p53 expression, tumor size, and peritumoral lymphatic vessel invasion are relevant prognostic markers in node-negative breast carcinoma. *J Clin Oncol* **12**: 454–466

Gasparini G, Toi M, Gion M, Verderio P, Dittadi R, Hanatani M, Matsubara I, Vinante O, Bonoldi E, Boracchi P, Gatti C, Suzuki H and Tominaga T (1997) Prognostic significance of vascular endothelial growth factor protein in node-negative breast cancer. *J Natl Cancer Inst* **89**: 139–147

Haagensen CD (1986) The relation of age to the frequency of breast carcinoma. In: *Disease of the Breast*, 3rd edn, Haagensen CD (ed); pp. 402–407, WB Saunders: Philadelphia

Harbeck N, Dettmar P, Thomssem C, Henselmann B, Kuhn W, Ulm K, Janicke F, Hofler H, Graeff H and Schmitt M (1998) Prognostic impact of tumor biological factors on survival in node-negative breast cancer. *Anticancer Res* **18**: 2187–2197

Horak ER, Leek R, Klenk N, Lejeune S, Smith K, Stuart N, Greenall M, Stepniewska K and Harris AL (1992) Angiogenesis, assessed by platelet/endothelial cell adhesion molecule antibodies, as indicator of node metastases and survival in breast cancer. *Lancet* **340**: 1120–1124

Isola JJ, Helin HJ, Helle MG and Kallioniemi O (1990) Evaluation of cell proliferation in breast carcinoma: comparison of Ki-67 immunohistochemical study, DNA flow cytometric analysis, and mitotic count. *Cancer* **65**: 1180–1184

Iwaya K, Tsuda H, Fukutomi T, Tsugane S, Suzuki M and Hirohashi S (1997) Histologic grade and p53 immunoreaction as indicators of early recurrence of node-negative breast cancer. *Jpn J Clin Oncol* **27**: 6–12

Kaplan EL and Meier P (1958) Non-parametric estimation from incomplete observations. *J Am.Stat Assoc* **53**: 457–481

Kato T, Kimura T, Muraki H, Miyakawa R, Tanaka S, Kamio T, Yamamoto K, Hamano K, Aiba M and Kawakami M (1996) Clinicopathological features associated with long-term survival in node-negative breast cancer patients. *Surg Today* **26**: 105–114

Kato T, Kimura T, Ishii N, Akiho F, Yamamoto K, Kameoka S, Nishikawa T and Kasajima T (1999) The methodology of quantitation of microvessel density and prognostic value of neovascularization associated with long-term survival in Japanese patients with breast cancer. *Breast Cancer Res Treat* **53**: 19–31

 © 2000 Cancer Research Campaign

Lampe B, Hantschmann P and Dimpfl T (1998) Prognostic relevance of immunohistology, tumor size and vascular space involvement in axillary node negative breast cancer. *Arch Gynecol Obstet* **261**: 139–146

Lauria R, Perrone F, Carlomagna C, De Laurentis M, Morabit A, Gallo C, Varriale E, Pettinato G, Panico L, Petrella G, Bianco AR and De Placido S (1996) The prognostic value of lymphatic and blood vessel invasion in operable breast cancer. *Cancer* **76**: 1772–1778

Lee AKC, Delellis RA, Silverman ML, Heatley GJ and Wolfe HJ (1990) Prognostic significance of peritumoral lymphatic and blood vessel invasion in node-negative carcinoma of the breast. *J Clin Oncol* **8**: 1457–1465

Mansour EG, Gray R, Shatila AH, Osborne CK, Tormey DC, Gilchrist KW, Cooper MR and Falkson G (1989) Efficacy of adjuvant chemotherapy in high-risk node-negative breast cancer. *N Engl J Med* **320**: 485–490

Mantel N (1966) Evaluation of survival data and two new rank order statistics arising in its consideration. *Cancer Chemother Rep* **50**: 163–170

Martin SA, Perez-Reyes N and Mendelsohn G (1987) Angioinvasion in breast carcinoma. An immunohistochemical study of factor VIII-related antigen. *Cancer* **59**: 1918–1922

McGuire WL, Tandon AK, Allred DC, Chamness GC and Clark GM (1990) How to use prognostic factors in axillary node-negative breast cancer patients. *J Natl Cancer Inst.* **82**: 1006–1015

Molina R, Segui MA, Climent MA, Bellmunt J, Albanelll J, Fernandez M, Filella X, Jo J, Gimenez N, Iglesias E, Miralles M, Alonso C, Peiro G, Perez-Pianol E and Ballesta AM (1998) p53 oncoprotein as a prognostic indicator in patients with breast cancer. *Anticancer Res* **18**: 507–511

O'Malley FP, Saad FZ, Kerkvliet N, Dolg G, Stitt L, Ainsworth P, Hundal H, Chambers AF, Turnbull DI and Bramwell V (1996) The predictive power of semiquantitative immunohistochemical assessment of p53 and c-*erb*B-2 in lymph node-negative breast cancer. *Hum Pathol* **27**: 955–963

Railo M, Lundin J, Haglund C, von Smetten K, von Boguslawsky K and Nordling S (1997) Ki-67, p53, Er-receptors, ploidy and S-phase as prognostic factors in T1 node negative breast cancer. *Acta Oncol* **36**: 369–374

Rosen PP, Groshen S, Saigo PE, Kinne DW and Hellman S (1989) A long-term follow-up study of survival in Stage I (T1N0M0) and Stage II (T1N1M0) breast carcinoma. *J Clin Oncol* **7**: 355–366

Saigo PE and Rosen PP (1987) The application of immunohistochemical stains to identify endothelial-lined channels in mammary carcinoma. *Cancer* **59**: 51–54

Sampat MB, Sirsat MV and Gangadharan P (1978) Prognostic significance of blood vessel invasion in carcinoma of the breast in women. *J Surg Oncol* **9**: 623–632

Sears HF, Janus C, Levy W, Hopson R, Creech R and Grotzinger P (1982) Breast cancer without axillary metastases. Are there high-risk biologic subpopulations? *Cancer* **50**: 1820–1827

Sitonen SM, Kallioniemi OP and Isola JJ (1993) Proliferating cell nuclear antigen immunohistochemistry using monoclonal antibody 19A2 and a new antigen retrieval technique has prognostic impact in archival paraffin-embedded node-negative breast cancer. *Am J Pathol* **142**: 1081–1089

Stemmermann GN, Catts A, Fukunaga FH, Horie A, Abraham M and Nomura Y (1985) Breast cancer in women of Japanese and Caucasian ancestry in Hawaii. *Cancer* **56**: 206–209

Tandon AK, Clark GM, Chamnes GC, Chirgwin JM and McGuire WL (1990) Cathepsin D and prognosis in breast cancer. *N Engl J Med* **322**: 297–302

van de Velde CJH, Gallager HS and Giacco GG (1986) Prognosis in node-negative breast cancer. *Breast Cancer Res Treat* **8**: 189–196

Weigand RA, Isenberg WM, Russo J, Brennan MJ and Rich MA (1982) The breast cancer prognostic study associates: blood vessel invasion and axillary lymph node involvement as prognostic indicators for human breast cancer. *Cancer* **50**: 962–969

Wright C, Angus B, Nicholson S, Sainsbury JRC, Cairns J, Gullick WJ, Kelly P, Harris AL and Horne CHW (1989) Expression c-*erb*B-2 oncoprotein: a prognostic indicator in human breast cancer. *Cancer Res* **49**: 2087–2090

Wynder EL, Kajitani T, Kuno J, Lucas JC, De Palo A and Farrow J (1963) A comparison of survival rates between American and Japanese patients with breast cancer. *Surg Gynecol Obstet* **117**: 196–200

*© 2000 Cancer Research Campaign*
